# Advancing climate change health adaptation through implementation science

**DOI:** 10.1016/S2542-5196(22)00199-1

**Published:** 2022-11-10

**Authors:** Gila Neta, William Pan, Kristie Ebi, Daniel F Buss, Trisha Castranio, Rachel Lowe, Sadie J Ryan, Anna M Stewart-Ibarra, Limb K Hapairai, Meena Sehgal, Michael C Wimberly, Leslie Rollock, Maureen Lichtveld, John Balbus

**Affiliations:** a Division of Cancer Control and Population Sciences, National Cancer Institute, Rockville, MD, USA; b Duke Global Health Institute and Environmental Science and Policy, Duke University, Durham, NC, USA; c Center for Health and the Global Environment, University of Washington, Seattle, WA, USA; d Climate Change and Health, Pan American Health Organization, Washington, DC, USA; e Global Environmental Health Program, National Institute of Environmental Health Science, Durham, NC, USA; f Barcelona Supercomputing Center (BSC), Barcelona, Spain; g Catalan Institution for Research and Advanced Studies (ICREA), Barcelona, Spain; h Centre on Climate Change and Planetary Health and Centre for Mathematical Modelling of Infectious Diseases, London School of Hygiene & Tropical Medicine, London, UK; i Department of Geography and the Emerging Pathogens Institute, University of Florida, Gainesville, FL, USA; j Inter-American Institute for Global Change Research (IAI), Montevideo, Uruguay; k Pacific Island Health Officers Association, Honolulu, HI, USA; l Environment and Health, The Energy and Resources Institute, New Delhi, India; m Department of Geography and Environmental Sustainability, University of Oklahoma, Norman, OK, USA; n Ministry of Health and Wellness, St Michael, Barbados; o Environmental and Occupational Health, University of Pittsburgh School of Public Health, Pittsburgh, PA, USA; p Global Environmental Health Program, National Institute of Environmental Health Science, Washington, DC, USA

## Abstract

To date, there are few examples of implementation science studies that help guide climate-related health adaptation. Implementation science is the study of methods to promote the adoption and integration of evidence-based tools, interventions, and policies into practice to improve population health. These studies can provide the needed empirical evidence to prioritise and inform implementation of health adaptation efforts. This Personal View discusses five case studies that deployed disease early warning systems around the world. These cases studies illustrate challenges to deploying early warning systems and guide recommendations for implementation science approaches to enhance future research. We propose theory-informed approaches to understand multilevel barriers, design strategies to overcome those barriers, and analyse the ability of those strategies to advance the uptake and scale-up of climate-related health interventions. These findings build upon previous theoretical work by grounding implementation science recommendations and guidance in the context of real-world practice, as detailed in the case studies.

## Background

As the urgency of the climate crisis escalates, with increasing morbidity and mortality from climate change-fuelled extreme weather and climate events, proactive and effective interventions to protect public health against the risks of a changing climate are crucial. Climate change-fuelled intensification of record-breaking heatwaves and wildfire seasons, recurrent severe hurricanes, severe flooding, and other climate-related exposures are causing mortality and exacerbating cardiovascular and respiratory illness, mental health disorders, and other climate-sensitive health outcomes. At the 2021 Conference of the Parties of the UN Framework Convention on Climate Change, 49 countries committed to the UN's 26th Climate Change Conference Health Programme to enhance the resilience of health systems through conducting national vulnerability and adaptation assessments and developing national and regional adaptation plans. Achieving climate-resilient and environmentally sustainable health systems requires evidence-based interventions to protect those at greatest risk.

The primary constraint to climate and health adaptation is the small amount of investment.^1^ Other constraints include identifying interventions that could effectively manage changes in the magnitude and pattern of climate-sensitive health outcomes. Early warning and response systems for heat-related mortality and a range of infectious diseases are crucial adaptation strategies, allowing health sectors to best manage health impacts due to temperature, precipitation, and other meteorological variables.^2^ Increasing the effectiveness of these strategies and similar interventions to best adapt and respond to current conditions will build capacity to manage additional climate change, within an iterative risk management framework that supports decision makers’ systematic responses to climate change while allowing for adjustments as new evidence emerges.^3^, ^4^ These processes and experiences afford a valuable opportunity to take stock of implementation challenges and to identify knowledge gaps to inform the design and successful deployment and uptake of adaptation interventions.

The field of implementation science has the potential to inform the development, scale-up, and evaluation of health adaptation efforts.^5^ Implementation science seeks to bridge the gap between research and practice and is rooted in action-oriented research.^6^ It is defined as the study of methods to promote the adoption and integration of evidence-based health interventions (eg, tools, programmes, and policies) into practice.^7^ Implementation science can optimise the development and use of evidence-based interventions, tools, guidelines, and programmes for complex public health challenges.^8^, ^9^ The field applies theories, frameworks, and models (herein collectively referred to as frameworks) to guide an understanding of determinants and processes that could influence the adoption, implementation, and sustainability of evidence-based interventions.^10^ Successful implementation accounts for contextual barriers and facilitators at multiple socio-ecological levels (eg, consumer and practitioner, organisation, community, municipality, region or state, and country) to support the adoption, integration, and sustainability of interventions.^11^ The field has described, developed, and tested a range of implementation strategies,^12^ the successes of which were determined by a range of outcomes including the acceptability, adoption, appropriateness, costs, feasibility, fidelity, penetration, and sustainability of the intervention.^13^

Increasing examples of implementation studies can provide empirical evidence to inform health adaptation efforts. This Personal View evolved from a Consortium of Universities for Global Health Conference satellite session in 2021. The goal of this paper is to highlight opportunities for which implementation science can enhance research efforts to develop and implement infectious disease early warning systems (EWSs) worldwide. We describe some of the common and context-based barriers to implementation in a set of five geographically diverse case studies that developed and implemented EWSs. Through retrospectively applying implementation science approaches to understanding these barriers, we make implementation science recommendations to guide health adaptation research that would advance the uptake and scale-up of these interventions.

## Methods

The five case studies were selected as a convenience sample to represent five different regions of the world that are disproportionately affected by climate change. These regions include Africa (Ethiopia), Asia (India), South America (Peruvian Amazon), Pacific Islands (Marshall Islands and Federated States of Micronesia), and the Caribbean (Barbados). None of the case studies were conducted using implementation science, but implementation science constructs were used retrospectively. Following the symposium, the authors met as a full group biweekly to discuss the case studies, learn key principles of implementation science, understand existing recommendations from Boyer and colleagues^5^ and how these recommendations applied to each of the cases, and identify key points that should be highlighted for each case study. Subsequently, each case study author met individually with an implementation science expert (GN) to discuss the specific case, the aims of the intervention in the case study, the specific barriers to its implementation, how these barriers were addressed in the case study, remaining challenges to implement and scale up the intervention, and how implementation science approaches could inform future efforts to implement and scale up such interventions. These individual conversations helped to frame the case study descriptions in the paper. Each case study author then described the key barriers they faced in implementing their systems, how they overcame those obstacles, and what challenges remain. Authors then reviewed the main implementation barriers across all five case studies and identified common challenges across the case studies. Subsequently, to show the value of implementation science frameworks, the authors selected and applied the Consolidated Framework for Implementation Research to understand how the identified barriers related to well established implementation science constructs that describe determinants of implementation. An experienced climate and environmental health scientist (WP) and an implementation science expert (GN) jointly reviewed the framework to come to a common understanding of its constructs. WP and GN then reviewed each case study in detail, mapping the challenges and successes to key domains of the framework via a deductive process. These mapping results were shared with and reviewed by case study investigators and discussed until concurrence was reached.

The implementation challenges identified collectively by the authors, along with the individual discussions among all the case study authors, served to create a set of recommendations of implementation science approaches to inform future efforts to implement and scale up these systems. These implementation science approaches can serve to optimise ongoing implementation and generate the necessary empirical evidence to inform future implementation and scale-up efforts.

## Case studies

Efforts to develop and implement EWSs and other tools based on environmental drivers are described. For each case study, the aims of the intervention, implementation challenges, how these challenges were addressed, and the remaining barriers to implementation and scale up are described. These common challenges were then categorised using the Consolidated Framework for Implementation Research, which characterises barriers and facilitators to enable effective implementation of interventions (ie, intervention characteristics, inner setting, outer setting, processes, and characteristics of individuals).^14^ A summary of implementation challenges across these studies is in the table. Although these case studies were not designed as implementation studies, they illustrate important lessons about deploying EWSs and guide recommendations for implementation science approaches that could inform future studies to develop, test, and scale-up these interventions.

### Case study one: malaria early warning scale-up in Ethiopia

Although there has been considerable progress towards malaria elimination in Ethiopia, a substantial disease burden remains, and resurgent epidemics are a concern.^15^ The goal of the Epidemic Prognosis Incorporating Disease and Environmental Monitoring for Integrated Assessment (EPIDEMIA) project is to develop malaria EWSs that can be used sustainably by public health institutions. These tools support routine forecasting of future malaria risk based on epidemiological surveillance and climate monitoring data.^16^, ^17^ There was a successful pilot implementation of EPIDEMIA in the Amhara region of Ethiopia.^18^ Formal engagement with the Ethiopian Federal Ministry of Health was conducted subsequently to evaluate the benefits and challenges of scaling malaria early warning to a national level; this project was supported by the United States Agency for International Development. Conducted in 2020, during the COVID-19 pandemic, the engagement involved virtual discussions with various stakeholder groups, interviews with key individuals, and an online survey.

A crucial lesson learned was that stakeholder engagement should have started from the beginning to achieve ownership of the malaria early warning process for sustainable implementation. Ethiopian stakeholders expressed a strong preference for running and maintaining the forecasting software tools.^19^ Substantial capacity building in data science will be required to achieve this goal. Malaria forecasts also need to be adapted to the multiple levels of Ethiopia's public health system.^19^ For example, national-level decision makers require long-lead forecasts for broad areas, whereas regional and local decision makers can act with shorter lead times but require higher geographical precision. The EPIDEMIA project has made considerable progress engaging with Ethiopian malaria experts from the outset.^18^ However, successful implementation and scale-up will require not only training and technology transfer but also a more symmetrical approach involving education, co-development, and long-term collaboration.

### Case study two: a dengue EWS in Barbados

In 2017, a national-level climate-driven dengue EWS was co-developed for Barbados, a small island state in the east of the Caribbean. The predictive model uses climate information to quantify the probability of dengue outbreaks several months in advance. The Barbados Ministry of Health and Wellness and the Barbados Meteorological Services are the implementation organisations, with support from the Caribbean Institute for Meteorology and Hydrology, the Caribbean Public Health Agency, and an interdisciplinary team of researchers.^20^, ^21^

In-person stakeholder engagement was crucial to establishing partnerships and the co-learning and co-development processes sustained by ongoing virtual meetings. Early on, climate and health stakeholders were interviewed to identify needs, perceptions, and current capacity to support a dengue EWS.^22^ A vital discovery of the modelling research was the non-linear and delayed effects of hydrometeorological extremes on dengue outbreaks.^23^ This finding emphasised the need for water-storage container management during drought events (eg, covering and cleaning containers and using larvicide) to avoid creating mosquito breeding habitats, which would increase the risk of dengue outbreaks. During a period of drought, the December, 2020 Caribbean Health Climatic Bulletin emphasised the importance of public health messages to encourage water-storage container maintenance; the bulletin was issued to ministries of health across the region.^23^, ^24^

The team is implementing the forecast tool on the online platform hosted by the national meteorological services, which issues routine climate services for other weather events (eg, dust and haze alerts). Key challenges included scarce capacity and resources to sustain these efforts, effective communication strategies, and an absence of formalised partnerships. Strategies to address these challenges included developing the most straightforward yet rigorous forecasting tool, convening workshops with stakeholders to co-develop and refine the model and visualisation according to stakeholder needs, and bringing stakeholders together to facilitate the cooperation needed for implementation.^25^

### Case study three: a malaria EWS in the Peruvian Amazon

Since 2011, the Amazon has had the largest percent increase in malaria cases compared with any other region in the world.^26^ A malaria early warning system (MEWS) was developed, with support from the USA's National Aeronautics and Space Administration (NNX15AP74G), that forecasts malaria outbreaks in administrative districts 12 weeks in advance and is being extended to Ecuador and Brazil through support from the USA's National Institute of Allergy and Infectious Diseases (R01AI151056). The MEWS uses near real-time hydrometeorological monitoring of climate and land cover data^27^ that is merged with government surveillance data, including census data and health intervention data. Statistical models forecast and visualise malaria incidence and provide probabilistic estimates for outbreak early warning.^28^, ^29^ In addition, an agent-based model is used to evaluate intervention strategies using synthetic population cohorts.^30^, ^31^, ^32^ Key stakeholders in the national and regional health systems were consulted throughout the process on all aspects of developing the MEWS, through qualitative interviews, workshops, and regional meetings, as well as establishing memorandums of understanding to formalise training and technology transfers.

Implementation challenges of the MEWS included: (1) rapid turnover of staff within the Peru Ministry of Health required re-engagement of key stakeholders; (2) no traditional use of environmental data to inform health decisions, nor previous experience or protocols for responding to early warnings; (3) limited training in geographical information systems and environmental science to understand and appropriately respond to model output; and (4) the decentralised health system requires multilevel engagement (eg, federal, region, and district), yet interlevel cooperation has become strained over time. Although the MEWS technology was intended to be directly transferred to government agencies, these challenges resulted in transferring the Land Data Assimilation System technology to academic partners in Ecuador (ie, to Universidad San Francisco de Quito) and Peru (ie, to Universidad Peruana Cayetano-Heredia), who will help expand MEWS forecasting to Ecuador and Brazil. Leveraging these academic partnerships with the government, the team conducted geographical information systems and statistical modelling workshops with ministry of health technical staff in 2021.

Additional important barriers to adoption were recognised that included overburdened technical staff, limited financial resources to incentivise adoption, and the need for capacity building in the areas of environmental science. By facilitating partnerships between local universities, intergovernmental organisations, and government, adoption capacities will be enhanced and will facilitate the identification of local champions.

### Case study four: EWSs for mosquito control in the Federated States of Micronesia and the Marshall Islands

Attempts to address dengue fever in the Federated States of Micronesia and the Marshall Islands have been highly reactive, relying solely on vector management during outbreaks. To reduce the effect and frequency of outbreaks, the Federated States of Micronesia and the Marshall Islands’ health ministries and the Pacific Island Health Officers Association are developing dengue EWSs to improve climate resilience using predictive models to forecast outbreaks, plan public health responses, and build vector management capacities. Major challenges include difficulty in querying the data and a skill shortage in data management of health workers, compounded by competing priorities set by management.

To address these challenges, step-by-step roadmaps are being used that include protocols on data gathering and processing for modelling, vector management, and outbreak preparedness. In addition, train the trainer events for the Federated States of Micronesia and the Marshall Islands environmental health workers will build skills and increase regional dissemination with the support of the Pacific Vector Management Council. The council was created in 2018, and is a coordination platform mandated by US-Affiliated Pacific Islands, including the Federated States of Micronesia and the Marshall Islands, and American Samoa, Guam, Northern Mariana Islands, and Palau.

A lesson learned is the need to scale up EWSs regionally to all US-Affiliated Pacific Islands because of the movement of people and diseases between jurisdictions. Going forward, stakeholder and community surveys will be conducted in the Federated States of Micronesia and the Marshall Islands and similar assessments with Pacific Vector Management Council members to identify aspects that could contribute to the effective implementation of EWSs in the region.

### Case study five: health vulnerability and adaptation assessment in India

A Health Vulnerability and Adaptation Assessment tool was developed by the case study team for India in 2019. The tool is a publicly available digital tool that ranks climate-linked health vulnerabilities by district. The tool uses 25 health-related indicators including climate-sensitive health outcomes (eg, the prevalence of nutritional deficiencies and vector-borne diseases), health infrastructure, socioeconomic status, and weather variables. Selection of indicators’ was based on a literature review and expert advice from representatives of departments of health and family welfare. The indicators are routinely collected from different government surveys at small area (ie, district) levels. The Health Vulnerability and Adaptation Assessment tool seeks to help reallocate financial resources towards investments in health care and preventive measures to areas for which climate risk-informed health-care training and infrastructure are needed the most.

The case study team partnered with the National Ministry of Health and Family Welfare to expand coverage of the tool across all 36 states and Union Territories. The team is partnering with governments of several states and the Union Territories across the country to guide them through the data collection and data assimilation processes. Successful deployment of this tool relies upon the ability of governments to reliably record the relevant indicators and provide them at appropriate temporal and spatial scales.

Deployment challenges include difficulty in collating data due, in part, to limited multisectoral collaboration between the public health, nutrition, and meteorology departments, as well as insufficient funding for deploying trained personnel and technological resources to process and transfer the data to the state governments. Additionally, special training is required to make the data from different departments compatible; for example, climate data are available on latitude–longitude grids whereas health indicators are available by administrative boundaries. Therefore, an exchange of domain knowledge and needs of different disciplines can facilitate scaling up.

To address these challenges, the project is building consortiums with diverse but complementary expertise (eg, disaster risk reduction, meteorology, and health), introducing project advisory committees with representatives from the government and cross-disciplinary experts, and inviting potential funders (eg, national representatives, bilateral organisations, and foundations) to project result dissemination webinars.

## Recommendations to advance implementation of EWSs

Together these five case studies show opportunities and constraints when developing and implementing EWSs (table). Key constraints that affect the uptake and sustainability of these EWSs include funding for sustainability and scale-up, local ownership, overburdened government workers, siloed government ministries, political instability, conflicting interests with the tourism sector, decentralisation, and issues related to capacity and infrastructure (eg, management of climate and health data, capacity in spatial data management, training in climate science for decision makers, and decision-support protocols).

**Table:** Key implementation challenges identified in the case studies

	**Description**	**Ethiopia**	**Barbados**	**Peruvian Amazon**	**Federated States of Micronesia**	**India**
Funding for sustainability	Internal or external funding to support partner participation in the development, testing, and implementation of climate-health tools and protocols; local partners often do not have budgets for engagement or some funding agencies (eg, the USA's National Aeronautics and Space Administration) prohibit funding to non-US entities, making engagement difficult	x	x	x	x	x
Funding for scaling	Climate-health early warning forecasting systems are often conducted in small regions or districts of a country to effectively design technical aspects of the system that can be used by community-based health workers or other decision makers; scaling up these tools and protocols to large geographical and political districts requires funding and evaluation	x	..	x	x	x
Local ownership	The adopting agency or institution taking control of the intervention, tool, or climate-health modelling responsibilities; ownership challenges include barriers in knowledge, time, and political commitment, among others	x	x	x	x	..
Overburdened government workers	The roles and responsibilities of government workers in low-income and middle-income countries might be defined equivalent to high-income country positions, but inefficiencies in government might result in tasks that require substantially more time (often double to triple the effort compared with high-income countries); asking for additional time for climate-health application adoption can be unrealistic	x	..	x	x	..
Multisectoral collaboration	Ministries are siloed as they receive independent funding to fulfil specific mandates that preclude cross-sectoral collaboration (ie, no mandates for health and climate; the degree to which an organisation is networked with other external organisations)	..	..	x	..	x
Political instability	Rapid turnover of elected officials or government political leadership; this turnover results in unstable government partnerships	..	..	x	..	..
Conflict with tourism sector	Creating a climate-health monitoring system that is publicly available can potentially affect the tourism sector by reducing travel during periods of high epidemic alert; this reduced demand can be damaging to local economies	..	x	..	..	..
Correlated problems, such as water scarcity	Communities face water scarcity, which is exacerbated by climate change, and creating solutions for one problem (ie, water storage containers) could exacerbate others (ie, creating larval habitats for mosquitoes) if containers are not properly managed	..	x	..	..	..
Decentralisation without a memorandum of understanding	Working with decentralised health systems requires strong relationships with each entity; if the memorandum of understanding is operating externally from the national health system, multiple memorandums could be required to obtain and share data	..	..	x	..	..
Data management (eg, climate and health data)	Managing environmental (eg, land, climate, and hydrology) and health (eg, surveillance) data requires sophisticated data skills and experience; skilled personnel are in short supply and great demand	x	x	x	x	x
Capacity training-data science, geographical information system	Knowledge of spatial data management and analysis is a rare, but increasing niche in epidemiology; this set of skills remains extremely rare	x	x	x	x	x
Climate science training among health decision makers	Climate science training is not a core competency in medical science, public health, or public policy; individuals in health-related decision-making positions are often naive to fundamental principles of climate science, reducing their capacity or willingness to make informed climate-related decisions	x	..	x	..	..
Decision-support protocols	Response to early warning forecasts requires well defined protocols to respond effectively; no country has a defined response to an early warning forecast for health outcomes	x	x	x	x	..

Drawing from these case studies, we recommend four implementation science approaches to conducting research on the development and deployment of an EWS (figure). These recommendations account for the different stakeholders, barriers, and constraints and offer approaches to addressing multisectoral stakeholder needs and overcoming implementation challenges. The recommendations include (1) applying implementation science frameworks to understand multilevel barriers and processes; (2) co-creating tailored implementation strategies with key stakeholders to overcome barriers; (3) analysing which, how, and why implementation strategies work; and (4) measuring implementation outcomes of the strategies analysed (eg, adoption, feasibility, fidelity, and sustainability; see subsequent sections). These recommendations are an iterative process. For example, upon analysing how and why strategies work, new barriers might become apparent that require iteration of the co-created strategies. Similarly, by measuring implementation outcomes, inadequacies in implementation efforts might become apparent, requiring additional application of frameworks to understand the barriers driving those inadequacies. Stakeholders (eg, implementing individuals and organisations, including communities and policy makers) are crucial throughout, across all four approaches. The success of these approaches relies on meaningful engagement, trust, and collaboration. Through explicit attention to understanding and overcoming barriers using community-centric and systems-driven methods, implementation science approaches can inform the most effective ways to ensure the adoption and sustained use of EWSs.

**Figure:**
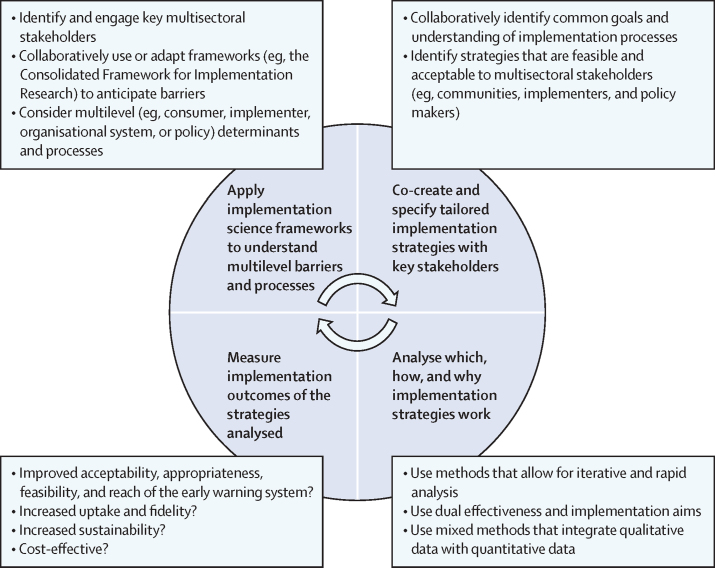
Recommendations for implementation science approaches in climate health adaptation research

## Recommendation one: apply implementation science frameworks to understand multilevel barriers and processes

A first step for successful implementation is to understand and anticipate potential barriers. As noted in the background section, the use of implementation science frameworks can help research teams, including key stakeholders, identify and anticipate potential barriers to implementation and consider multisectoral and multilevel (eg, consumer, implementer, organisational system, and policy) determinants and processes that can influence successful deployment. For example, the India case study illustrates how the development of the digital tool for ranking climate-linked health vulnerability in one Indian state faced challenges expanding to other states and Union Territories due to issues related to the intersectoral cooperation to access the needed data. And all the case studies noted a shortage of skilled personnel to manage and analyse data. Further, the case studies noted the absence of well defined health-related protocols to respond effectively to forecasts.

A plethora of implementation science frameworks have been developed to help guide the process of implementation, understand the influences on implementation, and evaluate implementation.^10^, ^11^ The Consolidated Framework for Implementation Research is one, of many, implementation science frameworks that could be adapted for EWSs to identify and understand determinants that influence implementation.^14^ For the purposes of this Personal View, we chose this framework as it is widely used in the field and the constructs are well operationalised. The determinants described in the Consolidated Framework for Implementation Research relate not just to the intervention itself but also the multilevel context in which the EWS is implemented.^14^ The framework constructs are categorised into: (1) characteristics relating to the EWS itself (eg, level of complexity, trialability, and relative advantage); (2) the implementers (eg, knowledge and beliefs, self-efficacy, state of change, and identification with organisation); (3) processes (eg, planning, engaging, executing, and evaluating); (4) the inner or organisational setting (eg, structural characteristics, networks and communication, culture, climate, and readiness for implementation); and (5) the outer or political setting (eg, needs and resources, cosmopolitanism, and external policies and incentives).^14^

Using the Consolidated Framework for Implementation Research facilitates the identification of barriers and constraints that could be generalised across settings. The panel shows that for a given constraint, there were a range of related determinants. For example, funding for sustainability relates to intervention costs and trialability, outer setting external policies and incentives, and inner setting organisational climate, relative priority, and readiness for implementation.

Panel **Implementation challenges from the case studies mapped on to the Consolidated Framework for Implementation Research constructs14**

**Funding for sustainability**

• Intervention: trialability and costs
• Inner setting: implementation climate and readiness for implementation
• Process: planning and engaging stakeholders
• Outer setting: external policies and incentives

**Funding for scaling**

• Intervention: costs, adaptability, and trialability
• Inner setting: implementation climate and readiness for implementation

**Local ownership**

• Inner setting: culture, relative priority, readiness for implementation, and tension for change
• Process: planning and engaging stakeholders

**Overburdened government workers**

• Inner setting: culture, implementation climate (eg, tension for change, organisational incentives, and learning climate), and readiness for implementation
• Process: planning and engaging stakeholders

**Multisectoral collaboration**

• Outer setting: cosmopolitanism (eg, the degree to which an organisation is networked with other external organisations)

**Political instability**

• Process: engaging leadership, champions, and external change agents

**Conflict with tourism sector**

• Outer setting: cosmopolitanism and peer pressure (eg, competitive pressure to implement an intervention)

**Correlated problems, such as water scarcity**

• Intervention: relative advantage and trialability
• Outer setting: external policy and incentives

**Decentralisation without a memorandum of understanding**

• Process: planning and engaging stakeholders
• Outer setting: external policy and incentives and cosmopolitanism

**Data management (eg, climate and health data)**

• Individuals: knowledge and beliefs, self-efficacy, and personal attributes
• Intervention: complexity
• Inner setting: learning climate and readiness for implementation (eg, access to knowledge and information)

**Capacity training-data science, geographical information system**

• Individuals: knowledge and beliefs, self-efficacy, and personal attributes
• Inner setting: learning climate and readiness for implementation (eg, access to knowledge and information)
• Outer setting: external policy and incentives

**Climate science training among health decision makers**

• Individuals: knowledge and beliefs, self-efficacy, and personal attributes
• Inner setting: learning climate and readiness for implementation (eg, access to knowledge and information)
• Outer setting: external policy and incentives
• Process: engaging stakeholders

**Decision-support protocols**

• Intervention: relative advantage, adaptability, and trialability
• Process: planning, engaging stakeholders, and executing

Implementation science frameworks can inform key barriers and processes to enable successful implementation. Through formal assessment of these potential barriers, stakeholders can identify key challenges to successful implementation and, thus, focus attention on developing strategies to overcome those barriers, as described in the next recommendation.

## Recommendation two: co-create and specify tailored implementation strategies with key stakeholders

After understanding barriers, a next crucial step is identifying strategies to overcome those barriers in collaboration with key stakeholders.^33^ The field of implementation science has identified and tested numerous strategies that promote the adoption and integration of evidence into practice. These include but are not limited to strategies to plan for implementation (eg, identifying key implementers), educate and train implementers to deliver an intervention, ensure those implementers and the systems in which they work have the needed support to incorporate the intervention into their workflow or to integrate the intervention into their community settings (eg, by providing technical assistance), support the fidelity of implementation, and inform the intervention and implementation efforts of needed adaptations to fit within a given context.^12^, ^34^

As shown in the case studies and is well understood within the field of climate adaptation,^35^ including a range of multisectoral stakeholders throughout developing and deploying adaptation options is crucial to successful implementation that is equitable and sustainable. These stakeholders include the individuals required to develop and operate the systems (eg, data analysts) and individuals required to mandate and support their use (eg, adopting agency officials, ministry officials, and health professionals). Including stakeholders from the beginning of a research project (ie, co-development of the intervention) can ensure the intervention is attentive to their needs and is acceptable, feasible, and equitably implemented. Thus, the figure includes cocreating and specifying strategies (eg, methods or techniques required to ensure the successful deployment) with key stakeholders to respond to identified multilevel barriers and constraints. This effort to specify strategies requires collaboratively engaging multisectoral stakeholders (eg, communities, implementers, and policy makers), building consensus on common goals and understanding implementation processes, and identifying feasible and acceptable strategies.

This value of engaging multisectoral stakeholders is illustrated by the Barbados case study of developing a dengue EWS, for which stakeholders identified safe water storage as crucial to address the problem. Stakeholder engagement across the case studies included workshops and meetings and the collection of qualitative and survey data (eg, Peruvian Amazon, Ethiopia, and Barbados), and helped ensure that implementation was equitable, developed in partnership with those most affected, reaching the populations most in need.

Strategies should be co-developed in response to the barriers identified through applying theory-informed frameworks and within the context of available resources and relevant stakeholders. Guidance on specifying and operationalising implementation strategies includes focusing on the following: the actor, the action, the action targets, the temporality, the dose, the implementation outcomes addressed, and the theoretical justification.^33^ The field has developed several approaches for codesigning and tailoring implementation strategies that include but are not limited to user-centred and human-centred design approaches, intervention and concept mapping, conjoint analysis, and simulation modelling.^36^, ^37^, ^38^, ^39^

For these and other approaches, the success of stakeholder engagement substantially depends on a transdisciplinary approach that incorporates experts from diverse disciplines with experts outside of academia (including community leaders and policy practitioners) who work together as equal team members to address a key issue and develop common project goals. Collaborative planning allows for changes during the project period and serves as a key foundation for sustainability. Project development and implementation depends on the collective identification of feasible and acceptable strategies that can be sustained over time.

## Recommendation three: analyse which, how, and why implementation strategies work

Upon identifying promising strategies to overcome barriers, the next step would be to analyse their successes. There are a range of analytic approaches that can support future implementation studies of EWSs to understand which strategies work best where, and how and why they work. These approaches include iterative and adaptive study designs^40^ and rapid-cycle research approaches to allow for incremental and contextually informed modifications,^41^ and the use of mixed methods that integrate qualitative with quantitative data^42^ to understand the reasons for those modifications. Given the ongoing development and refinement of EWSs, studies with a dual focus on EWS effectiveness and deployment efforts could be warranted. These hybrid effectiveness–implementation designs combine effectiveness research with implementation research and are often used when the effectiveness of the intervention being studied is uncertain.^43^ For example, in a study testing the predictive value of an EWS, investigators can also collect data on factors that might influence successful implementation, such as understanding readiness to implement or implementation conditions.^14^

In studies that seek to test different sets of strategies to implement an EWS, for which the outcome is a measure of implementation efforts (eg, feasibility, fidelity, or sustainability), and the unit of analysis is a region or country, a quasi-experimental design might be feasible and appropriate. Examples of these include stepped wedge, interrupted time series, and pre-designs and post-designs with a non-equivalent control group.^40^, ^44^

Studies that seek to understand not just whether an implementation strategy worked, but how or why it did or did not, could benefit from the use of qualitative or mixed methods research. The latter is a mixing of quantitative and qualitative approaches in which the qualitative approaches can provide more in-depth understanding of the quantitative observations, or conversely, can help to shape the quantitative approaches.^42^ The case studies from Ethiopia, Barbados, and the Peruvian Amazon all exemplify the value of qualitative (eg, stakeholder interviews) and quantitative (eg, online survey) data to inform the implementation of the EWS. Understanding how and why strategies work can ensure that strategies are appropriately selected to target specific barriers to implementation.^45^

## Recommendation four: measure implementation outcomes of the strategies analysed

To analyse the success of strategies to improve implementation, measures of implementation success must be delineated and assessed. As defined in the background section, implementation outcomes focus on how the intervention (ie, the EWS) is implemented in a given setting versus if the intervention worked (ie, was effective). Therefore, our last recommendation is to measure implementation outcomes after applying these strategies to assess their success, as measured by increased acceptability, appropriateness, feasibility, uptake, fidelity, sustainability, and cost-effectiveness of the EWS.^13^ Collecting data on these implementation outcomes would provide important information to inform potentially needed adaptations of the implementation strategy or the EWS itself and inform the scale-up and spread of the EWS to other contexts.

The five case studies highlight areas for which data could be collected. For example, the Peruvian case study illustrates how the rapid turnover of members within the Ministry of Health affected the feasibility of the malaria EWS in this setting. Evaluating how well an implementation strategy helped address this feasibility barrier could improve uptake of the EWS. The Barbados case study highlights how their strategy of engaging stakeholders early and through workshops can improve the acceptability of the EWS. Sustainability also emerged as a key issue in these case studies. The case studies highlighted how the sustainability of the EWSs were jeopardised by a scarcity of funding and knowledge needed to run the EWSs. Collecting these data are also vital to best understand how to scale up these interventions. For example, the Ethiopia case study illustrates how the sustainability of the malaria EWS is in peril due to the scarcity of local stakeholder capacity in data science to use the interventions. Fostering local ownership can help ensure investment in the needed capacity to support the uptake and sustainability of the EWS.

The field of implementation science has developed numerous resources to assist in defining and measuring implementation outcomes.^46^, ^47^ Given the relative nascence of the field, there are many opportunities to further develop and refine these measures as relevant to EWSs.^48^

## Limitations

Although the five cases highlight both unique and common barriers to implementing EWSs, they do not represent all possible scenarios and settings in which EWSs would be implemented. And thus, these barriers are not an exhaustive list and could miss other crucial barriers to implementing EWSs. Additionally, none of the case studies were conducted using implementation science methods, but implementation science constructs were used retrospectively. However, the recommendations we offer are broadly relevant to advance the implementation of EWSs.

## Conclusion

Understanding the best ways to implement EWSs can be broadly relevant to address a range of current crises. From the COVID-19 pandemic to climate change to the threat of nuclear war, effective EWSs can provide not only the needed forecasts, but also the range of effective response options and the time to increase disaster preparedness at all scales. To ensure effective deployment of EWSs, the scientific community can build a knowledge base on how best to anticipate and overcome barriers and constraints.

Despite case study successes in cocreation of EWSs, the barriers identified highlight areas ready for implementation science to inform adoption and sustained use of EWSs. For example, what implementation strategies can best address the issue of a scarcity of knowledge and skills to deploy EWSs? Implementation science has shown that training alone is insufficient. To ensure fidelity and sustained use, implementers could require technical assistance and quality management (eg, audit and feedback) strategies to integrate new knowledge into their workflows. What implementation strategies can best facilitate multisectoral buy-in, partnership building, and collaboration? Implementation mapping with a range of stakeholders has been shown to build these relationships and needed buy-ins.^36^ What implementation strategies can support the scale-up of EWSs? How can environmental and climate science and practice be more effectively integrated into public health and clinical practice and policy? These are examples of research questions that if addressed through implementation approaches could enhance resilience to a changing climate.

Multilevel contextual challenges require multilevel strategies. The case studies illustrate societal and structural-level contextual factors that impede implementation, such as political instability and infrastructure issues. Implementation science provides a valuable set of approaches to evaluate how effective implementation strategies could be leveraged to address multilevel barriers in global settings.

## Declaration of interests

DFB is a staff member of the Pan American Health Organization. The author alone is responsible for the views expressed in this publication, and they do not necessarily represent the decisions or policies of the Pan American Health Organization. LKH was funded in part by a grant to the Pacific Island Health Officers Association from the US Department of State (Cooperative Agreement Number SLMAQM20CA2490). The opinions, findings and conclusions stated herein are those of the authors and do not necessarily reflect those of the US Department of State. All other authors declare no competing interests.
